# Three-week schedule of irinotecan plus cisplatin in patients with previously untreated extensive-stage small-cell lung cancer

**DOI:** 10.1038/sj.bjc.6603500

**Published:** 2006-11-28

**Authors:** Y S Hong, H R Lee, S Park, S C Lee, I G Hwang, B-B Park, J Lee, J S Ahn, M-J Ahn, H Y Lim, K Park

**Affiliations:** 1 Division of Hematology-Oncology, Department of Medicine, Samsung Medical Center, Sungkyunkwan University School of Medicine, Seoul, Korea

**Keywords:** three-week schedule, irinotecan, cisplatin, extensive-stage small-cell lung cancer

## Abstract

Irinotecan and cisplatin demonstrated promising outcomes in extensive-stage small-cell lung cancer. According to the dosage and schedule of irinotecan, efficacy and toxicity profiles showed subtle differences. This study was designed to evaluate efficacy and toxicity of 3-week schedule of irinotecan/cisplatin in patients with previously untreated extensive-stage small-cell lung cancer. The primary objective was to evaluate response rate and secondary objectives were overall survival and progression-free survival. Patients with previously untreated extensive-stage small-cell lung cancer were enrolled. Irinotecan 65 mg m^−2^ was administered on days 1 and 8 and cisplatin 60 mg m^−2^ on day 1. Treatment was repeated every 3 weeks. Seven out of 54 patients (13.0%) had complete response, and partial response was observed in 33 (61.1%). The overall response rate was 74.1% (95% CI; 62.0–82.2%). Stable disease was observed in eight (14.8%) and no progressive disease was observed. After a median follow-up duration of 28.7 months, the median overall survival and progressive-free survival were 13.6 and 6.5 months, respectively. Major grade 3/4 toxicities were neutropenia (50.0%), anorexia (42.6%), diarrhoea (29.6%), fatigue (29.6%) and vomiting (13.0%). There was one treatment-related death owing to pneumonia. Three-week schedule of irinotecan/cisplatin showed effective antitumour activity and moderate toxicities in patients with previously untreated extensive-stage small-cell lung cancer.

Lung cancer is the leading cause of cancer-related deaths in Western countries (Jemal *et al*, 2004), and also in Korea (Shin *et al*, 2004). The proportion of histologic diagnosis with small-cell lung cancer (SCLC) in the United States is approximately 15% of patients with lung cancer (Jemal *et al*, 2004), and more than half of these patients are diagnosed with extensive-stage disease. Incidence of SCLC had paralleled trends in cigarette smoking, and smoking prevalence for the adult population is relatively high in Korea. During the last decade, most patients with extensive-stage SCLC (ES-SCLC) have been treated with etoposide and platinum resulting in a median survival of 8–10 months (Roth *et al*, 1992). Although several trials of platinum-based combination chemotherapy were performed, it failed to show a superiority to combination of etoposide and cisplatin (EP) (Mavroudis *et al*, 2001; Sundstrom *et al*, 2002).

Irinotecan, which inhibits the function of topoisomerase I in cancer cells, demonstrated synergism and non-cross resistance when combined with platinum agents (Fukuda *et al*, 1996; Masuda *et al*, 1996). Kudoh *et al* (1998) reported that combination chemotherapy of irinotecan and cisplatin (IP) in patients with SCLC showed a promising response rate of 84 with 29% of complete responses and median survival over 13 months. Since then, several phase II/III trials of irinotecan-based combination chemotherapy with different dose and schedule were performed with varying results in SCLC patients (Noda *et al*, 2002; Kudoh *et al*, 2005; Hanna *et al*, 2006; Kinoshita *et al*, 2006; Schmittel *et al*, 2006). In a previous phase III trial conducted by a Japanese group, patients were treated with 60 mg m^−2^ of cisplatin on day 1 and 60 mg m^−2^ of irinotecan on days 1, 8, and 15 every 4 weeks, and showed a good median survival of 12.8 months. However, only 80.4% of planned dose of irinotecan was administered and the day-15 irinotecan was omitted in 50% of patients owing to toxicities (Noda *et al*, 2002). Another phase III trial performed in the West tested 65 mg m^−2^ of irinotecan on days 1 and 8 every 3 weeks in ES-SCLC patients, and showed rather lower median survival of 9.3 months (Hanna *et al*, 2006) compared with the Japanese trial, suggesting possible ethnic and pharmacogenomic differences between the two study populations.

Based on these results, we conducted a phase II study using irinotecan 65 mg m^−2^ on days 1 and 8, and cisplatin 60 mg m^−2^ on day 1 schedule in chemotherapy-naïve ES-SCLC patients to evaluate the efficacy and safety of the regimen in Korean patients.

## PATIENTS AND METHODS

### Patients' eligibility

Patients with previously untreated ES-SCLC were enrolled. Patients were eligible if they had (1) histologically confirmed small-cell carcinoma; (2) extensive-stage disease; (3) Eastern Cooperative Oncology Group (ECOG) performance status (PS) of ⩽3; (4) age over 18 years old; (5) adequate haematologic parameters (haemoglobin ⩾9.0 g dl^−1^, absolute neutrophil count ⩾1500 *μ*l^−1^, platelet count ⩾100 000 *μ*l^−1^), renal functions (serum creatinine ⩽1.5 mg dl^−1^ or calculated creatinine clearance by Cockroft formula ⩾50 ml min^−1^), and hepatic function (aspartate aminotransferase, alanine aminotransferase ⩽3 × upper limits of normal, total bilirubin ⩽2 × upper limits of normal); (6) at least one bi-dimensionally measurable lesion according to the WHO criteria (Miller *et al*, 1981); (7) absence of active infection; (8) no prior chemotherapy, radiotherapy, or surgery for the disease; (9) no history of myocardial infarction in the last 6 months before the study entry; (10) no uncontrolled congestive heart failure or significant arrhythmia; and (11) no prior second primary cancer, except for cervix cancer *in situ* or skin cancer. Patients with brain metastases were allowed provided that there were no significant neurologic symptoms or signs. All patients provided written informed consent before they entered the study. This protocol was approved by the institutional review board at Samsung Medical Center.

### Treatment and dose modification

Patients were treated with irinotecan 65 mg m^−2^ on days 1 and 8, and cisplatin 60 mg m^−2^ on day 1. Treatment cycles were repeated every 3 weeks until the maximum six cycles initially planned, documented disease progression, unacceptable toxicity, or patient's refusal. Cisplatin was given with adequate hydration of 2 l of intravenous fluid to protect renal functions and with diuretics if needed to control volume status. Cholinergic symptoms that occurred during or within 1 h after irinotecan administration were treated with atropine (0.3 mg or as needed). Loperamide was provided as therapy for prophylaxis of delayed diarrhoea. A 5-hydroxytryptamine type-3 receptor antagonist was given as emesis prophylaxis before drug administration.

Application of chemotherapy was delayed by one or two weeks until haematologic recovery of absolute neutrophil count ⩾1500 *μ*l^−1^ and platelet ⩾100 000 *μ*l^−1^. Administration of irinotecan was omitted on day eight if grade 2 or 3 diarrhoea occurred, and subsequent cycles were allowed when the diarrhoea recovered to baseline or grade⩽1. The dose of irinotecan in subsequent cycles was reduced by 25% from the planned dose if there were any grade 4 hematologic toxicities lasting more than 7 days, ⩾grade 3 haematologic toxicities with febrile episode, or bleeding-associated thrombocytopenia. Treatment was discontinued in patients with grade 4 diarrhoea. The dose of cisplatin in subsequent cycles was reduced by 25% from the planned dose if there were grade 4 haematologic toxicities or if grade 2 renal toxicity. In the presence of grade 3 or 4 non-haematologic toxicity (except nausea, vomiting, and alopecia), the treatment was postponed until resolution of the toxicity and then both drug doses were reduced by 25% for the next cycle. Once a dose reduction was required, re-escalation of dose was not allowed.

### Assessment of efficacy and toxicity

The following pretreatment evaluations were performed within 2 weeks before study entry: a full medical history and physical examination, complete blood cell count with differentials, chemistry profiles, urinalysis, and performance status evaluation. Chest X-rays, chest and upper abdominal computed tomography (CT) scans, brain magnetic resonance imaging, radionuclide bone scan and any other diagnostic procedures as clinically indicated were performed within 4 weeks before enrollment. During treatment, a limited history taking, physical examination, assessment of toxicity, complete blood cell count with differentials, blood chemistry and chest X-rays were performed every 3 weeks before each cycle. Appropriate imaging studies including CT scans of chest and upper abdomen were performed every two cycles (6 weeks) to assess treatment response, and sooner if needed for documentation of disease progression. Objective tumour responses were assessed according to the WHO criteria (Miller *et al*, 1981). Complete response (CR) was defined as the disappearance of all known disease for at least 4 weeks with no new lesion appearing. Partial response (PR) referred to an at least 50% decrease in the sum of the products of bidimensional diametres lasting for at least 4 weeks without the appearance of new lesions. Stable disease (SD) was defined as failure to observe a PR or CR, no progressive or new lesions were observed for at least 4 weeks. Progressive disease (PD) was defined as a 25% or greater increase in the sum of the products of bidimensional diameters of any measurable lesion or the appearance of new lesions. All enrolled patients were included in the intention-to-treat analysis of efficacy. Response rate was calculated as the ratio of the number of patients who achieved complete or partial responses to the number of enrolled patients. Overall survival (OS) and progression-free survival (PFS) were calculated from the start of therapy until death and progression, respectively, or until last follow-up. Toxicities were monitored according to the National Cancer Institute Common Toxicity Criteria (NCI-CTC) scale version 3.0.

### Statistical consideration

The sample size was calculated according to Simon's two-stage optimal design (Simon, 1989). A targeted objective response rate of 80% versus an objective response rate of no interest of 60% with a power of 0.90 at a one-sided significance level of 0.05 was chosen, and accrual of 45 assessable patients was projected. In the initial stage, 18 evaluable patients were to be entered into the study and evaluated for response. If there were > 11 responses, accrual was to be terminated. If ⩾11 responses were observed in the first stage, then 27 additional patients were to be entered in the second stage to achieve a target sample size of 45 evaluable patients. Assuming that 10% of patients were inassessable, at least a total of 50 patients were planned to be accrued for this study. All patients who received at least one course of therapy were considered assessable for toxicity, and all eligible patients who received at least one cycle of therapy were included for survival estimation. Descriptive statistics were reported as proportions and medians. OS and PFS were assessed by the Kaplan–Meier method and the 95% confidence interval (95% CI) for the median time to event was computed.

## RESULTS

### Patient characteristics

Between November 2002 and January 2005, 54 patients were enrolled and their clinical characteristics are shown in Table 1. The median age was 64 years (range, 47–78 years), 49 patients (90.7%) were male and five (9.3%) were female. Forty patients (74.1%) had ECOG PS of 0–1, whereas 14 patients (25.9%) had ECOG PS of 2 or 3. The most common sites of metastasis were lymph nodes (40/54, 74.1%) and brain metastasis was observed in 17 patients (31.5%) at the time of enrollment. Median serum lactate dehydrogenase (LDH) level at initial diagnosis was 408 IU l^−1^ (range, 225–1566).

### Drug administration

Drug administration and relative dose intensity are shown in Table 2. The median number of cycles to be administered was four (range 1–6 cycles), and 24 (44.4%) patients completed the planned six cycles of chemotherapy. Treatment was delayed for a median of 2 weeks in 109 out of subsequent 223 cycles (48.9%). The most common cause for delayed administration was neutropenia (58 cycles, 53.3%). Twenty-two cycles (9.9%) required dose reductions mainly due to neutropenia and diarrhoea. The delivered dose intensity (DI) was 17.4 mg m^−2^ week^−1^ (87.0% of planned dose) for cisplatin and 36.6 mg m^−2^ week^−1^ (84.5% of planned dose) for irinotecan.

### Response

Forty-eight (88.9%) of 54 patients were evaluable for responses. Six patients were not evaluable, but were included in the intent-to-treat analysis. Two patients refused to receive chemotherapy after first cycle of the regimen and were lost to follow-up, one patient was referred to another hospital, one patient was dropped out after first dose of the regimen owing to poor performance status, one patient underwent operation for gastric ulcer perforation, and there was one treatment-related death.

The overall response rate was 74.1% (95% CI; 62.0—82.2%), with a complete response rate of 13% (Table 3). SD was observed in eight patients (14.8%) on initial assessment of response.

### Survival

**Figure 1:**
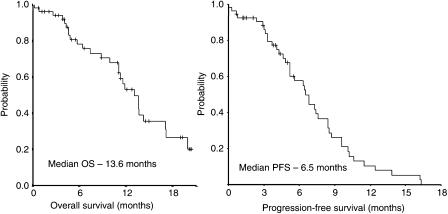
OS and PFS of the all patients.

All 54 patients were included in the survival analysis on an intent-to-treat basis. The OS and PFS of patients in this study is shown in Figure 1. After the median follow-up duration of 28.7 months (range, 10.9–45.0 months), the median overall survival was 13.6 months (95% CI, 10.7–15.5 months) and 1 year survival rate was 53.1%. The median PFS was 6.5 months (95% CI, 5.1–7.9 months) and 1 year PFS was 10.5%.

### Toxicities

All patients were evaluable for toxicities, and grade 3 or 4 toxicities observed during treatment are listed in Table 4. Haematologic toxicity was most prevalent in this study. Grade 3 or 4 neutropenia occurred in 27 (50.0%) of 54 patients. Grade 3 or 4 anaemia and thrombocytopenia were observed in two (3.7%) of 54 patients, respectively. Most significant ⩾grade 3 non-haematologic toxicity was anorexia (*n*=23, 42.6%). Grade 3 or 4 diarrhoea occurred in 16 (29.6%) patients, with grade 2 diarrhoea in seven (13.0%) patients and grade 1 diarrhoea in 22 (40.7%) patients. In addition, 24 (44.4%) patients showed grade 2 alopecia and seven (13.0%) showed grade 2 neuropathy. There was one treatment-related death. That patient was a 51-year-old male with ECOG performance status score of 3, and had brain metastasis at initial presentation of disease. He developed neutropenic sepsis with pneumonia caused by *Streptococcus pneumoniae* on day 19 and died of aggravation of pneumonia on day 26 of the first cycle of chemotherapy despite recovery from neutropenia.

### Salvage chemotherapy

Of 55 patients, 24 patients (43.6%) were treated with systemic chemotherapy as second-line treatment, and 22 patients were evaluable for treatment responses. Salvage regimens included etoposide and carboplatin (11/24, 45.8%), EP (10/24, 41.7%), oral etoposide and carboplatin (2/24, 8.3%), and paclitaxel and ifosfamide (1/24, 4.2%). By intent-to-treat analysis, overall response rate of salvage treatment was 37.5% (95% CI, 18.1–56.9%) with one CR and eight PR. SD and PD were observed in four patients (16.7%) and nine patients (37.5%), respectively. Median time to progression (TTP) was 3.4 months (1.0–20.0 months) in all 24 patients and median duration of response was 3.6 months (1.6–19.8 months) in responders. We defined chemo-sensitive relapse as PD was noted after 3 months of maximal response, and primary refractory disease as initial maximal response was SD, or PD was noted within 3 months of maximal response. As our definition, 17 patients (17/24, 70%) showed chemo-sensitive relapse, and seven patients (7/24, 30%) showed primary refractory disease. In 17 patients with chemo-sensitive relapse, responses to second-line chemotherapy were observed in five patients (29.4%, with one CR and four PR.), SD in three patients (17.6%), and PD in seven patients (41.2%). Two of 17 patients with chemo-sensitive relapse were inevaluable. In seven patients with primary refractory disease, response to second-line chemotherapy was observed in four patients (57.1%, with zero CR and four PR), SD in one patient (14.3%), and PD in two patients (28.6%). Response differences in these two groups did not show statistical significance (*P*=0.531).

## DISCUSSION

For treatment for extensive-stage SCLC, combination chemotherapy with EP has been the standard regimen since the last decade. However, combination chemotherapy with IP came into the spotlight after the Japanese phase II trial in 1998 (Kudoh *et al*, 1998). In 1998, Kudoh *et al* (1998) reported 84% of overall response rate with 29% of complete response and 13 months of median OS in patients with extensive-stage SCLC. In this trial, irinotecan was administrated at 60 mg m^−2^ on days 1, 8 and 15 repeated every 3 weeks. In this dose and schedule of irinotecan, grade 3 or 4 haematologic toxicities were exceedingly frequent with 77% of neutropenia, 45% of leucopenia. Despite high incidence of haematologic toxicities, this study was the first to report median survival that exceeds 1 year in ES-SCLC (Kudoh *et al*, 1998).

The promising preliminary results led to several irinotecan-based phase II and III trials in patients with SCLC. Two most representative phase III trials were published in 2002 and 2006, respectively (Noda *et al*, 2002; Hanna *et al*, 2006). In 2002, Noda *et al* (2002), reported that combination with irinotecan and cisplatin (IP) was superior to that of EP in terms of response rates and OS (65%, 12.8 months *vs* 52%, 9.4 months, *P*=0.002) for the patients with ES-SCLC. Although IP seemed more effective than EP, the toxicities were higher in the IP arm with 65.3% of the patients with grade 3/4 neutropenia and 26.7% with grade 3/4 diarrhoea. In this trial, irinotecan was administered with 60 mg m^−2^ on days 1, 8 and 15 every 4 weeks in combination with 60 mg m^−2^ of cisplatin on day one, and the relative dose intensity was 80.4% of the planned dose. Most recently, however, Hanna *et al* (2006) reported IP did not show survival benefit compared with EP (9.3 *vs* 10.2 months, *P*=0.74). This trial adopted 65 mg m^−2^ of irinotecan on days 1 and 8 every 3 weeks schedule in combination with 30 mg m^−2^ of cisplatin on day 1 and 8, and showed lower overall response rate of 48% and shorter median OS of 9.3 months compared with previous trial of Noda *et al* (2002), in IP group. The toxicities seemed significantly lower than previous trial with only 36.2% of patients experiencing grade 3/4 neutropenia in IP group (Hanna *et al*, 2006).

In this study, irinotecan was administered at 65 mg m^−2^ on days 1 and 8, combined with 60 mg m^−2^ of cisplatin on day 1 in a 3-week schedule; thus, the dosages would be in the medium range as compared to those used in previous two trials. This regimen showed 74.1% of response rate and 13.6 months of median OS in the same patient population. It surpasses the results of Kudoh *et al* (1998) in median OS over 13 months, and showed higher response rates than that of equal dosage of irinotecan (Hanna *et al*, 2006). These results are very encouraging and superior to other trials with similar irinotecan dosages and schedules. Grade 3/4 toxicities were 50% in neutropenia and 29.6% in diarrhoea, and were comparable with previous other trials (Kudoh *et al*, 1998; Noda *et al*, 2002; Hanna *et al*, 2006; Kinoshita *et al*, 2006). However, one treatment-related death was observed in this trial. In the present study, four patients (7.4%) had initial poor performance status of ECOG score 3 (Table 1). One patient died during chemotherapy as previously noted, another two patients could not complete the planned treatment owing to toxicities. Only one patient could receive scheduled chemotherapy and showed response of stable disease. As the SCLC is one of the most chemosensitive tumour types, patients with poor performance status (PS ⩾3) who are not usually included in clinical trials of other tumour types had often been included in some trials. The present study also included a few patients with poor PS who, however, did not do so well with this treatment as mentioned above. Therefore, special cautions need to be exerted for patients with poor performance status in future trials. Given the considerable toxicities and the median number of cycles to be administered was four cycles, one may also need to consider to shorten the treatment cycles to four from six in future trials.

Irinotecan is metabolised by carboxylesterase to an active metabolite 7-ethyl-10-hydroxycamptothecin (SN-38). SN-38 is then further metabolised in the liver by uridine diphosphate-glucuronosyltransferases (UGTs) to an inactive metabolite, SN-38 glucuronide (SN-38G) (Mathijssen *et al*, 2001). This glucuronidatioin is the major route of detoxification of irinotecan, thus inherited differences in irinotecan metabolism may have an important influence on the pharmacokinetics and toxicity of this drug. Han *et al* (2006) reported the UGT1A polymorphisms could predict treatment outcomes and toxicities of irinotecan in Korean patients. In this report, patients with homozygote of UGT1A1^*^6 allele had shorter PFS and more irinotecan-related toxicities. Six of 81 Korean patients (7.4%) had phenotype of UGT1A1^*^6 homozygosity. Another phenotype associated with low tumour responses and high irinotecan-related toxicity is UGT1A1^*^28, and it is highly prevalent in Caucasian individuals with reported frequencies of 0.29–0.47 (Bosma *et al*, 1995; Premawardhena *et al*, 2003), whereas it has much lower frequency in Asians (0.08–0.19) (Yamamoto *et al*, 1998). In Korean report, the frequency of UGT1A1^*^28 was 0.07 (Han *et al*, 2006). Therefore, it could be explained that ethnic differences might be involved in discrepant results in terms of efficacy and toxicity of irinotecan.

Given the similar schedule and dosage of irinotecan and cisplatin combination chemotherapy in the trial performed by Hanna *et al* (2006) compared with our study, higher response rate (74.1 *vs* 48%) and longer median survival (13.6 *vs* 9.3 months) was observed in our study. However, grade 3 or 4 toxicities were more common in our study; neutropenia (50 *vs* 36.2%), febrile neutropenia (14.9 *vs* 3.7%), and diarrhoea (29.6 *vs* 21.3%). Even though the difference in dose and administration schedule, as well as disease characteristics might be plausible explanations of the disparities of outcome and toxicities between the two trials, it might be largely attributable to the pharmacogenomic/ethnic differences as was previously discussed by Hanna *et al* (2006), Further study with UGT1A polymorphism in this population will be needed.

In conclusion, our results suggest that 3-week schedule of irinotecan (65 mg m^−2^, on days 1 and 8) and cisplatin (60 mg m^−2^, on day 1) showed comparable results in terms of response rate, survival, and toxicity with the 4-week schedule of JCOG. Considering toxicity and convenience, 3-week schedule of irinotecan and cisplatin can be a reasonable option in the treatment of patients with ES-SCLC in Korea, which, however, requires further study in Caucasian population.

## Figures and Tables

**Table 1:** Patient characteristics

	**No. of patients**	**%**
**Characteristics**	**54**	**100**
Age, years (range)	Median 64 (47–78)	
		
*Sex*		
Male	49	90.7
Female	5	9.3
		
*ECOG PS*		
0	2	3.7
1	38	70.4
2	10	18.5
3	4	7.4
		
*No. of distant metastatic sites*		
0	9	16.7
1	20	37.0
2	14	25.9
⩾3	11	20.4
		
*Metastatic sites*		
Lymph nodes	40	74.1
Brain	17	31.5
Bone	16	29.6
Pleural effusion or seeding	16	29.6
Liver	14	25.9
Adrenal glands	11	20.4
Initial LDH (IU l) levels	Median 408 (225–1566)	

ECOG=Eastern Cooperative Oncology Group; LDH=lactate dehydrogenase; PS=performance status.

**Table 2:** No. of chemotherapy cycles and delivered actual dose

**Cycles**	**No. of patient (%)**	
1	7 (13.0)	
2	9 (16.7)	
3	4 (7.4)	
4	8 (14.8)	
5	2 (3.7)	
6	24 (44.4)	
	**Delivered dose/planned dose (mg m^−2^ week^−1^)**	**% of planned dose**
Cisplatin	17.4/20.0	87.0
Irinotecan	36.6/43.3	84.5

**Table 3:** Objective responses

**Tumour response (*n*=54)**	**No. of patients**	**%**
Complete response*	7	13.0
Partial response*	33	61.1
Stable disease	8	14.8
Progressive disease	0	0
Not evaluable	6	11.1

* Overall response rate: 74.1% (95% CI, 62.0–86.2).

**Table 4:** Toxicity profiles

	**Grade 3**		**Grade 4**	
	**No. of patients**	**%**	**No. of patients**	**%**
*Hematologic toxicities*				
Neutropenia	18	33.3	9	16.7
Thrombocytopenia	0	0.0	2	3.7
Anaemia	2	3.7	0	0.0
Febrile neutropenia	7	13.0	1	1.9
				
*Nonhematologic toxicities*				
Anorexia	22	40.7	1	1.9
Diarrhoea	15	27.7	1	1.9
Fatigue	15	27.7	1	1.9
Nausea/vomiting	7	13.0	0	0.0
Stomatitis	5	9.6	0	0.0
Constipation	4	7.4	0	0.0
